# 巨噬细胞抑制因子-1与早期非小细胞肺癌诊断及预后相关性

**DOI:** 10.3779/j.issn.1009-3419.2016.04.05

**Published:** 2016-04-20

**Authors:** 宇宁 刘, 小兵 王, 腾 王, 超 张, 坤鹏 张, 若川 臧, 修益 支, 伟 张, 克林 孙

**Affiliations:** 100021 北京，北京协和医学院中国医学科学院肿瘤医院胸外科 Department of Toracic Surgery, Detection Center of Tumor Biology, Cancer Institute and Hospital, Chinese Academy of Medical Sciences and Peking Union Medical College, Beijing 100021, China

**Keywords:** 肺肿瘤, 巨噬细胞抑制因子-1, 肿瘤标志物, 预后, Lung neoplasms, Macrophage inhibitory cytokine-1, Tumor biomarker, Prognosis

## Abstract

**背景与目的:**

巨噬细胞抑制因子-1（macrophage inhibitory cytokine-1, MIC-1）是人转化生长因子β（transforming growth factor-β, TGF-β）超家族中重要成员，研究发现MIC-1表达水平在多种上皮来源肿瘤患者血清中均有显著升高。本研究旨在探讨MIC-1在早期非小细胞肺癌（non-small cell lung cancer, NSCLC）诊断及其与临床病理特征间的关系，以及与术后复发/转移及预后的相关性。

**方法:**

采用酶联免疫吸附试验（enzymelinked immunosorbent assay, ELISA）方法检测152例早期肺癌、48例肺良性疾病患者及105例正常对照人群血清MIC-1浓度，分析MIC-1诊断肺癌中的作用，同时分析血清MIC-1浓度与临床病理特征、复发/转移及预后的相关性。

**结果:**

早期肺癌患者组MIC-1血清水平高于正常对照组（*P* < 0.001）和肺良性疾病组（*P* < 0.001），设1, 000 pg/mL为诊断肺癌的临界值，MIC-1检测肺癌的敏感性和特异性分别为70.4%和99.0% [曲线下面积（area under curve, AUC）: 0.90；95%CI: 0.87-0.94]；MIC-1血清水平与年龄（*P*=0.001）、性别（*P*=0.03）有关，病理TNM分期T2的患者MIC-1血清水平高于T1患者（*P*=0.022）；血清MIC-1 > 1, 465 pg/mL组的患者3年生存率为77.6%，低于血清MIC-1 < 1, 465 pg/mL组的患者94.8%（*P*=0.022），*Cox*回归多因素分析结果显示，血清MIC-1 > 1, 465 pg/mL是Ⅰ期、Ⅱ期NSCLC独立的预后因素（HR=3.37, 95%CI: 1.09-10.42, *P*=0.035）。

**结论:**

MIC-1作为血清肿瘤生物标志物，可能有助于提高肺癌早期诊断。MIC-1的检测对判断Ⅰ期、Ⅱ期NSCLC患者预后有预测价值，可能为其独立的预后指标。

## Abstract (en)

**Background and objective:**

Increased macrophage inhibitory cytokine-1 (MIC-1), member of transforming growth factor-β (TGF-β) superfamily, was found in patients serum with epithelial tumors. Therefore, our aim was to delineate the diagnostic and prognostic value of serum MIC-1 in patients with stage Ⅰ-Ⅱ non-small cell lung cancer (NSCLC).

**Methods:**

A total of 152 consecutive patients with stage Ⅰ-Ⅱ NSCLC were prospectively enrolled and underwent follow up after total resection of tumor. Serum MIC-1 level was detected in lung cancer patients by ELISA, 48 benign pulmonary disease patients and 105 healthy controls, and was correlated with clinical features and prognosis of patients.

**Results:**

The level of MIC-1 of NSCLC patients was significantly higher than that of controls (*P* < 0.001) and benign pulmonary disease patients (*P* < 0.001). A threshold of 1, 000 pg/mL could be used to diagnose early-stage NSCLC with 70.4% sensitivity and 99.0% specificity. The level of MIC-1 was associated with elder age (*P*=0.001), female (*P*=0.03) and T2 (*P*=0.022). A threshold of 1, 465 pg/mL could identify patients with early poor outcome with 72.2% sensitivity and 66.1% specificity. The overall 3-year survival rate in patients with high level of MIC-1 (≥1, 465 pg/mL) was significantly lower than that of patients with low MIC-1 level (77.6% *vs* 94.8%). Multivariable *Cox* regression revealed that a high level of MIC-1 was an independent risk factor for compromised overall survival (HR=3.37, 95%CI: 1.09-10.42, *P*=0.035).

**Conclusion:**

High level of serum MIC-1 could be served as a potential biomarker for diagnosis and poorer outcome in patients with early-stage NSCLC.

非小细胞肺癌（non-small cell lung cancer, NSCLC）是一种发病率居恶性肿瘤首位且预后较差的恶性肿瘤，手术是Ⅰ期-Ⅲa期NSCLC患者最重要的治疗手段，对Ⅱ期和Ⅲ期NSCLC患者术后常规采用铂类辅助化疗可改善预后^[1-3]^；然而，目前Ia期和Ib期NSCLC的术后辅助治疗仍然存在争议^[4]^。早期（Ⅰ期、Ⅱ期）NSCLC患者在接受手术治疗后，仍有约40%-50%在术后出现了局部复发或/和远处转移，5年总生存率为60%-90%^[5-9]^。TNM分期是评价NSCLC病变累及程度的重要手段^[10]^，然而分期特征相近的部分患者其预后仍可能存在差异^[11]^。因此，在早期NSCLC中，对可能出现复发或转移的高危患者筛选出来，在此基础上指导更积极的全身和局部治疗，可能对延长患者的生存时间具有重要意义。

巨噬细胞抑制因子-1（macrophage inhibitory cytokine-1, MIC-1），属于转化生长因子β（transforming growth factor-β, TGF-β）超家族，参与人体内组织急性损伤后的炎症反应和组织修复等。MIC-1表达水平在多种上皮来源肿瘤患者血清中均有提高，且与肿瘤的发病机制密切相关。在肿瘤细胞中，如颅内脑肿瘤、黑色素瘤、肺癌、胃肠、胰腺癌、结直肠癌、前列腺癌和乳腺癌，MIC-1的表达水平升高。MIC-1细胞因子可能为肿瘤的发展提供多种作用，并可能参与了肿瘤的增殖、迁移、转移和肿瘤细胞耐药^[12]^。此外，临床数据显示MIC-1蛋白过度表达提示患者预后较差。因此，MIC-1细胞因子可能成为一种新的生物标志物并用于肿瘤的诊断和预后的判断，并成为新的治疗靶点。

本研究应用自主研发的MIC-1血清检测试剂盒对单中心规范治疗的早期肺癌患者进行研究，探讨MIC-1在肺癌早期诊断中的应用价值，研究MIC-1血清水平与临床病理特征的关系，并评价MIC-1血清水平对Ⅰ期和Ⅱ期NSCLC患者预后的预测价值，为早期NSCLC的诊断筛查和个体化治疗提供新的血液检查参考指标。

## 1 资料与方法

### 1.1 临床资料

本研究方案经中国医学科学院肿瘤医院伦理委员会批准，经过患者知情同意。研究选取2011年9月-2013年5月于中国医学科学院肿瘤医院胸外科行原发性肺癌根治性切除加系统性淋巴结清扫，术后经病理科细胞学及组织病理学确诊，并根据国际抗癌联盟（Union for International Cancer Control, UICC）第七版肺癌肿瘤-淋巴结-转移（tumor-node-metastasis, TNM）分期，明确肿瘤无外侵、无远处纵隔淋巴结转移及远处转移的早期NSCLC成年患者（18岁以上），术后根据国家癌症综合治疗网络（National Comprehensive Cancer Network, NCCN）NSCLC治疗指南进行治疗；排除①影像学资料不完整，无法明确临床分期；②经腹部计算机断层扫描（computed tomograhy, CT）/B超，头部核磁/CT，骨扫描等疑诊或确诊转移癌或合并其他恶性肿瘤；③既往有肿瘤病史或术前接受过放疗化疗等辅助治疗；④不能依从研究方案完成随访的病例共152例，以及肺良性疾病48例，正常对照105例，良性疾病患者经中国医学科学院肿瘤医院病理科细胞学或组织病理学诊断，正常人群血样来自中国医学科学院肿瘤医院健康体检人群，入选标准为体格检查、血清学肝肾功能检查及影像学结果均为隐性者。入组患者入院后在所有治疗开始前抽全血2 mL，分离血清后冻存（-80 ℃）。患者的临床信息及血清标本检测结果由未直接参与诊疗及检测过程的医生采集并录入结构化数据库，在血清标本采集及标志物检测过程中对患者的诊断和病理资料实行盲法。

### 1.2 血清肿瘤相关抗原标志物检测及结果判定

试剂盒：采用中国医学科学院肿瘤医院生物检测中心研发，北京金紫晶生物技术有限公司生产的酶联免疫吸附试验（enzymelinked immunosorbent assay, ELISA）试剂盒；血清MIC-1测定：测血清检测操作过程严格按照试剂盒使用说明书进行^[13]^。吸光度与校准品和血清样品中的MIC-1浓度呈正比，可通过剂量-反应曲线得出样品中的MIC-1浓度。临界参考值：MIC-1的临界参考值为1, 000 pg/mL。

### 1.3 术后随访

术后每半年随访患者，完成胸部增强CT，并根据临床情况进行腹部CT/B超，头部核磁/CT，骨扫描等检查，记录肿瘤复发或转移的部位及时间，以及患者死亡的原因及时间。随访资料通过门诊复查、电话及通信方式获得。

### 1.4 统计学分析

应用SPSS 22.0软件进行统计分析，计量资料经方差齐性检验后，对符合方差齐性的两组独立样本计量资料比较，采用独立样本*t*检验；多组独立样本比较，采用方差分析；不符合正态分布计量资料采用非参数检验。计数资料比较采用卡方检验；受试者工作特征（receiver operating characteristic, ROC）曲线用于评价血清MIC-1对早期NSCLC诊断和预后评估的价值；生存资料描述，以肿瘤死亡作为主要结局事件，肿瘤复发或转移作为次要结局事件。生存期从手术之日起计算，终点日期为主要结局事件发生时间或末次随访时间（2015年11月），采用*Kaplan-Meier*法描绘生存曲线，并进行*Log-rank*检验比较组间差异；*Cox*比例风险回归模型用以评价血清MIC-1水平对术后生存期的影响；*P* < 0.05为差异有统计学意义。

## 2 结果

### 2.1 研究人群

本研究共纳入术后病理明确诊断的早期NSCLC患者共152例，其中女性89例（58.5%），男性63例（41.5%），年龄27岁-79岁，平均年龄58.8岁。Ⅰ期患者98例（64.47%），Ⅱ期患者54例（35.53%），腺癌116例（76.32%），鳞癌36例（23.68%），高分化、高-中分化38例（25%），中分化、中低分化、低分化114例（75%），肿瘤直径 < 3 cm者105例（69.1%），肿瘤直径≥3 cm者47例（30.9%）。肿瘤患者均经组织病理学诊断且采血时未接受过任何治疗。肺良性疾病48例，其中男性20例（41.67%），女性28例（58.33%），年龄23岁-75岁，平均年龄53.2岁；病理诊断：炎症18（37.5%）例，结核13（27.08%）例，错构瘤8（16.67%）例，硬化性血管瘤6（12.5%）例，腺瘤样增生2（4.17%）例，囊肿1（2.08%）例。正常对照组105名，来自中国医学科学院肿瘤医院，男性59例（56.3%），46例（43.81%），年龄20岁-79岁，平均年龄51.4岁，均无消化系统疾病，肝肾功能正常。经人口统计学基线数据比较，肺良性疾病组与正常对照组以及肺癌组患者的年龄，性别进行比较，均无统计学差异。

### 2.2 血清MIC-1蛋白水平诊断早期NSCLC的价值

肺癌组分别与肺良性疾病组、正常对照组比较，NSCLC患者外周血血清中位MIC-1蛋白水平为1, 346.28 pg/mL，高于肺良性疾病组（848.12 pg/mL）与正常对照组（367.46 pg/mL），差异具有统计学意义。肺癌患者、肺良性疾病患者与正常人群血清中MIC-1水平见表 1，肺癌组分别与肺良性疾病组和正常对照组比较，两组间MIC-1水平有差异（*P* < 0.001）。

**1:** 肺癌、肺良性疾病组与正常对照组血清MIC-1水平 The serum level of MIC-1 of NSCLC patients, lung benign disease patients and control

Group	*n*	P25 (pg/mL)	P50 (pg/mL)	P75 (pg/mL)	*P*
Lung cancer	152	940.50	1, 324.50	1, 819.00	< 0.001
Lung benign disease	48	667.07	848.12	1, 239.80	
Controls	105	213.07	367.46	592.59	
NSCLC: non-small cell lung cancer; MIC-1: macrophage inhibitory cytokine-1.					

以肺癌组和正常对照组MIC-1浓度绘制ROC曲线。MIC-1诊断肺癌的ROC曲线下面积（Area Under Curve, AUC）为0.90（95%CI: 0.87-0.94）（图 1A），综合考虑ROC曲线和正常对照人群MIC-1水平，设1, 000 pg/mL为诊断肺癌的临界值，MIC-1检测肺癌的敏感性和特异性分别为70.4%和99.0%。

**1:**
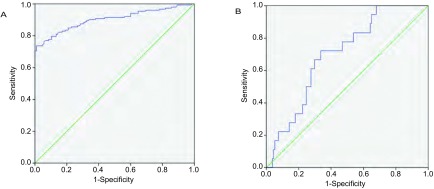
NSCLC患者和健康对照者血清MIC-1水平受试者工作曲线（ROC）（A）；早期NSCLC术后3年是否死亡的患者血清MIC-1水平ROC曲线（B）。 The ROC curve of serum MIC-1 for the diagnosis of lung cancer (A); The ROC curve of serum MIC-1 level for predicting postoperative outcomes within 3 years after surgery survival (B). ROC: receiver operating characteristic curve.

### 2.3 血清MIC-1水平与早期NSCLC患者临床病理特征的关系

MIC-1血清水平可能与年龄（*P*=0.001）、性别（*P*=0.03）以及术后早期（3年内）死亡结局有关（*P*=0.042）（表 2）。女性、60岁及以上的老年患者中MIC-1血清水平明显增高。尽管不同肿瘤T分期的MIC-1水平无差异，但进一步两组间比较提示T2期的肿瘤患者MIC-1水平较T1期增高（*P*=0.022）（图 2）。现有资料未发现MIC-1水平与肿瘤直径、病理类型、分化程度、分期、淋巴结状态以及早期NSCLC术后3年内发生复发/转移等有关（*P* > 0.05）。

**2:** 不同临床病理特征的早期NSCLC患者血清MIC-1水平 Comparison of the serum MIC-1level among different pathological characteristics (pg/mL)

Characteristics	*n*	Mean (pg/mL)	SD	*P*
Age (year)				0.001
< 60	64	1, 221.39	838.21	
≥60	88	1, 665.85	809.71	
Gender				0.030
Female	89	1, 603.85	708.35	
Male	63	1, 301.92	725.07	
Tumor size (cm)				0.200
< 3	105	1, 426.85	887.49	
≥3	47	1, 594.57	748.84	
Smoking history (year)				0.060
< 20	83	1, 361.11	925.53	
≥20	69	1, 620.17	726.11	
Histologic type				0.458
Squamous cell	36	1, 579.20	729.58	
Adenocarcinoma	116	1, 446.51	910.47	
Stage				0.141
Ⅰ	98	1, 034.80	814.33	
Ⅱ	54	1, 615.24	897.99	
Differentition				0.184
Moderate-well	38	1, 320.18	1, 057.10	
Poor	114	1, 531.55	764.29	
T				0.066
T1	42	1, 223.90	648.02	
T2	101	1, 585.81	924.241	
T3	9	1, 465.89	512.00	
N				0.193
N0	124	1, 430.26	798.13	
N1	24	1, 674.54	1, 460.50	
N2	4	1, 880.66	1, 600.10	
Recurrence/metastasis				0.270
No	118	1, 438.48	865.92	
Yes	34	1, 618.32	779.32	
Survival information				0.042
Alive	134	1, 427.60	851.50	
Dead	18	1, 859.22	736.98	

**2:**
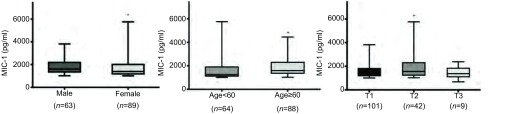
不同临床病理特征的血清MIC-1水平。女性患者（*P* =0.001），60岁及以上的老年患者（*P* =0.03）的血清MIC-1水平显著较高，T2期的肿瘤患者MIC-1水平较T1期显著增高（*P* =0.022）。 The level of MIC-1 was higher in patients who were female (*P* =0.001), ≥60-year-old (*P* =0.03) and T2 stage (*P* =0.022) at diagnosis.

### 2.4 血清MIC-1水平与早期NSCLC预后的关系

研究入组152例患者，总随访时间4年，中位随访时间为37.61个月。随访期内无失访病例，18例（11.84%）出现主要结局事件（肿瘤所致死亡），总体3年生存率88.16%；34例（22.36%）出现次要结局事件（复发或转移），其中局部复发7例（4.6%），发生远处转移27例（17.76%）。

随访期内死亡的患者治疗前血清MI C -1水平（1, 859.22±736.98 pg/mL）明显高于未出现死亡的患者（1, 427.60±851.50 pg/mL）（*P*=0.042）（表 2）。以早期NSCLC术后3年内是否出现主要结局事件的患者血清MIC-1蛋白水平做ROC（图 1B），AUC为0.70（95%CI: 0.59-0.81），根据ROC曲线，当临界值为1, 465 pg/mL，其预测患者术后随访期内不良预后的灵敏度为72.2%，特异度为66.1%。

以MIC-1=1, 465 pg/mL对本组患者进行分组（表 3），相比于血清低MIC-1水平组（MIC-1 < 1, 465 pg/mL），血清高MIC-1水平组（MIC-1≥1, 465 pg/mL）的早期NSCLC患者以女性、老年患者多见（*P* < 0.01）；长期吸烟者相对更多，但尚无统计学差异；肿瘤直径≥3 cm者（*P*=0.011），T分期（*P*=0.034）及临床分期更高（*P*=0.026），随访期内出现死亡的患者也明显增多（22.4% *vs* 5.3%, *P*=0.002）。

**3:** 不同MIC-1水平的早期NSCLC患者临床病理特征 The baseline and follow-up of patients with different serum MIC-1 level [*n* (%)]

Factors	MIC < 1, 465 pg/mL (*n*=94)	MIC≥1, 465 pg/mL (*n*=58)	Total (*n*=152)	*P*
Clinical characteristic				
Gender				0.001
Female	49 (52.1%)	40 (69.0%)	89 (58.5%)	
Male	45 (47.9%)	18 (31.0%)	63 (41.5%)	
Age (year)				< 0.001
< 60	53 (56.4%)	11 (19.0%)	64 (42.1%)	
≥60	41 (43.6%)	47 (81.0%)	88 (57.9%)
Smoking history (year)				0.057
< 20	57 (60.6%)	26 (44.8%)	83 (54.6%)	
≥20	37 (39.4%)	32 (55.2%)	69 (45.4%)	
Tumor size (cm)				0.011
< 3	72 (76.6%)	33 (56.9%)	105 (69.1%)	
≥3	22 (23.4%)	25 (43.1%)	47 (30.9%)	
Histologic type				0.210
Squamous cell	19 (20.2%)	17 (29.3%)	36 (23.7%)	
Adenocarcinoma	75 (79.8%)	41 (70.7%)	116 (76.3%)	
Differentition				0.102
Moderate-well	28 (29.8%)	10 (17.2%)	38 (25.0%)	
Poor	66 (70.2%)	48 (82.8%)	114 (75.0%)	
T				0.034^*^
T1	32 (34.0%)	10 (17.2%)	42 (27.6%)	
T2	57 (60.6%)	44 (75.9%)	101 (66.5%)	
T3	5 (5.3%)	4 (6.9%)	9 (5.9%)	
N				0.156^*^
N0	80 (85.1%)	44 (75.9%)	124 (81.6%)	
N1	12 (12.8%)	12 (20.7%)	24 (15.8%)	
N2	2 (2.1%)	2 (3.4%)	4 (2.6%)	
Stage				0.026
Ⅰ	67 (71.3%)	31 (53.4%)	98 (64.47%)	
Ⅱ	27 (28.7%)	27 (46.6%)	54 (35.53%)	
Follow-up infomation				
Survival information Survival information				0.002
Alive	89 (94.7%)	45 (77.6%)	134 (88.16%)	
Dead	5 (5.3%)	13 (22.4%)	18 (11.84%)	
Recurrence/metastasis				0.107
No	77 (81.9%)	41 (70.7%)	118 (77.63%)	
Yes	17 (18.1%)	17 (29.3%)	34 (22.36%)	
^*^Mann-Whitney U test.				

*Kapan-Meier*生存曲线显示：血清MIC-1≥1, 465 pg/mL的患者3年生存率为77.6%，低于血清MIC-1 < 1, 465 pg/mL的患者（94.8%）（图 3），血清高MIC-1水平组术后出现主要结局（死亡）事件更早（*Log-rank*, *P*=0.002）；血清低MIC-1水平组中80%的主要结局事件出现于术后12个月-18个月间，而血清高MIC-1水平组的主要结局事件出现于术后5个月-25个月间，提示对两组的患者可能需要不同的随访策略。

**3:**
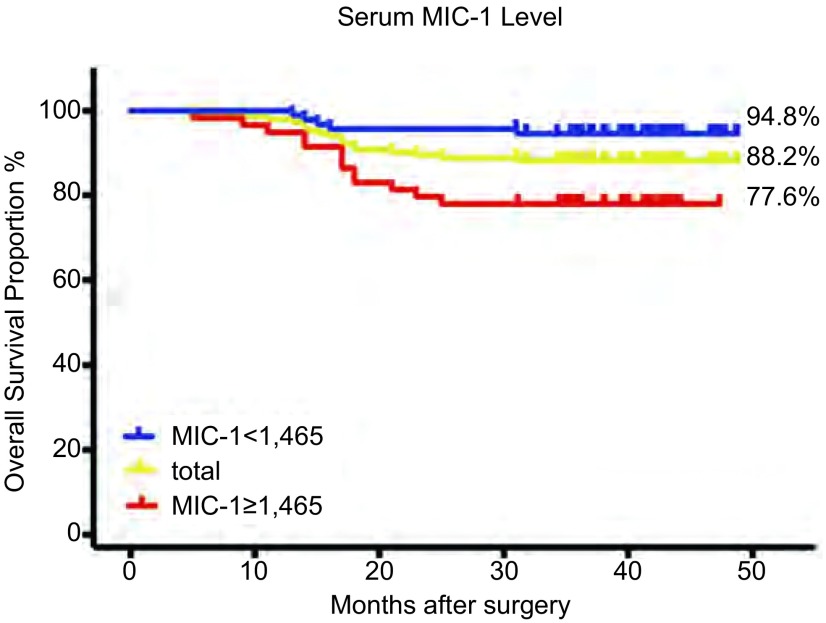
血清MIC-1 < 1, 465 pg/mL、MIC-1≥1, 465 pg/mL与总生存期的生存曲线 Overall survival curves and 3-year survival rate of patients with high level of MIC-1 (< 1, 465 pg/mL) and low level of MIC-1 (≥1, 465 pg/mL).

单因素*Cox*生存分析提示高血清MIC-1患者的死亡风险是低MIC-1组的4.56倍（HR=4.56, 95%CI: 1.62-12.79, *P*=0.004），进一步排除性别、年龄、长期吸烟史、肿瘤大小、肿瘤T分期等与血清MIC-1水平有潜在关系的因素影响，多因素*Cox*比例风险回归模型提示高血清MIC-1水平是患者术后死亡的独立危险因素（HR=3.37, 95%CI : 1.09-10.42, *P*=0.035）（表 4）。

**4:** 早期NSCLC患者术后生存期与预测因素的关系 Association of potential predictors with overall survival after surgery for patients with early stage NSCLC

Item	B	SE	Wald	*P*	HR	95%CI for HR
MIC-1	1.211	0.576	4.429	0.035	3.370	1.090-10.420
T	-	-	6.675	0.036	-	-
T1	0.723	1.107	0.427	0.514	2.060	0.235-18.035
T2	2.512	1.251	4.034	0.045	12.332	1.063-143.120
Gender	-0.280	0.697	0.161	0.688	0.756	0.193-2.964
Age	1.036	0.722	2.062	0.151	2.819	0.685-11.598
Smoking	-0.269	0.606	0.197	0.657	0.764	0.233-2.504
Tumor size	1.122	0.587	3.657	0.056	3.070	0.972-9.694

## 3 讨论

肺癌是对人类健康和生命威胁最大的恶性肿瘤之一。在中国，不到30年间肺癌相关死亡率增长了464.84%^[14]^，这可能是由吸烟增长以及空气污染加剧所导致^[15]^。2012年，国家癌症中心收集了全国104个癌症登记处提交的2009年肿瘤登记情况的报告，对其中符合标准的72个登记处的数据分析显示肺癌的发病率和死亡率均居首位。目前肺癌治疗的一个不能令人满意的现状是多数患者在治疗后仍有复发和转移，即使手术切除，手术后患者体内仍会有亚临床转移灶的存在，即使行辅助化疗，由于肿瘤细胞的凋亡过程受到抑制，仍然难以避免肿瘤复发。影像学检查、支气管镜等在肺癌的诊断、复发检测方面具有重要的价值，是临床常用的医学手段，但往往不便于在大规模筛查中使用。大量研究和防治资料证实早期诊断和早期治疗是防治与降低死亡率的最有效办法，肿瘤标志物的水平与肿瘤的发生、发展、消退、复发等有良好的相关性，因此长期以来寻找能早期诊断并判断预后的肿瘤标志物成了人们关注的焦点。

MIC-1是近年发现的肿瘤标志物，作为TGF-β家族的成员，广泛参与细胞凋亡、侵袭及转移等生物学过程，而究竟是促进还是抑制取决于肿瘤的病理类型。正常人群血清中MIC-1蛋白呈低水平稳定表达，在肿瘤状态下表达水平显著升高。本研究应用自主研发的双抗体夹心ELISA血清MIC-1检测试剂盒，对肺癌患者及肺良性疾病患者和正常人群进行了血清学研究，发现肺癌患者血清MIC-1水平显著高于正常人群（*P* < 0.001）和肺良性疾病患者（*P* < 0.001），提示MIC-1蛋白可能在肺癌的发生、发展过程中发挥作用，成为一种新的诊断NSCLC的肿瘤标志物。将血清MIC-1临界值设定为1, 000 pg/mL时，其诊断肺癌的敏感性和特异性分别为70.4%和99.0%，表明MIC-1在肺癌的早期诊断中具有重要的应用价值，有可能成为比较理想的肺癌诊断及筛查标志物。肺癌筛查降低了肿瘤患者发现就为晚期的风险。虽然用低剂量计算断层摄影筛查出早期肿瘤患者，降低肺癌死亡率，但存在假阳性的可能^[16, 17]^。因此，具有较高特异性血清MIC-1水平可辅助LDCT提高发现早期肺癌的可能。

对早期NSCLC患者的MIC-1血清水平研究表明：女性MIC-1血清水平高于男性、60岁及以上的老年患者MIC-1血清水平高于中青年患者，提示MIC-1蛋白可能与年龄、性别相关；病理TNM分期T2的患者MIC-1血清水平高于T1患者，提示MIC水平可能与早期肺癌局部侵袭有关。吸烟的患者血清MIC-1水平较不吸烟患者有增高趋势，可能吸烟是影响血清MIC-1的因素。本研究中，早期（Ⅰ期、Ⅱ期）的NSCLC患者中MIC-1血清水平与肿瘤直径、病理类型、分化程度、分期、淋巴结状态等无明显相关性。

对患者预后的进一步研究提示MIC-1可能作为生物学标志物用于判断早期NSCLC患者的预后。血清MIC-1≥1, 465 pg/mL组的患者3年生存率仅为77.6%，低于血清MIC-1 < 1, 465 pg/mL组的患者94.7%，进一步排除年龄、性别、长期吸烟史、肿瘤大小、肿瘤局部侵袭程度（T分期）等因素对结果的潜在影响，发现术前血清高MIC-1水平（≥1, 465 pg/mL）是Ⅰ期-Ⅱ期NSCLC患者术后死亡的独立危险因素，因此术前血清MIC-1可能能够对对筛选早期肺癌术后预后较差的高危患者有重要意义；且相比于其他有创检查和分子病理学的检测手段，血清指标的评估具有简单、快速、可重复等优点，可能有助于术前了解患者危险分层，指导治疗方案选择及强化辅助治疗；另外，不同MIC-1水平组的患者在术后出现死亡的时间也存在差异，提示低MIC水平组患者应强调术后18个月内的随访，而高MIC-1水平组患者更需要连续定期随访。未来的研究可进一步验证高危患者是否确实能够从血清MIC-1检测指导治疗中获益。

由于本研究人群为早期NSCLC患者，但对早期患者的辅助治疗和强化治疗仍存在争议，术后辅助治疗有可能部分存在差异，由于本研究为单中心研究，治疗方案主要参照NCCN NSCLC治疗指南，仅少数患者接受了术后辅助治疗，且纳入患者时间跨度较短，因此可能部分反映实际情况，但由于本研究未对术后辅助治疗与预后的关系进行分析，因此研究结果对其他患者群体的应用尚需更多研究探索。

综上所述，血清MIC-1水平的检测对预测Ⅰ期、Ⅱ期NSCLC手术治疗预后可能有重要的临床参考意义，血清MIC-1高水平可能是患者预后不良的一种表现。对于预后不良的高危Ⅰ期、Ⅱ期NSCLC术后患者，多学科综合治疗措施可能使其获益，从而提高Ⅰ期、Ⅱ期NSCLC患者的整体治愈率。然而，在血清MIC-1水平在被纳入临床检测之前，仍需要大样本的前瞻性研究进一步评估其应用价值。
